# Investigation of the Antineoplastic Effects of 2-(4-Chlorophenyl)-13α-Estrone Sulfamate against the HPV16-Positive Human Invasive Cervical Carcinoma Cell Line SiHa

**DOI:** 10.3390/ijms24076625

**Published:** 2023-04-01

**Authors:** Hazhmat Ali, Péter Traj, Gábor J. Szebeni, Nikolett Gémes, Vivien Resch, Gábor Paragi, Erzsébet Mernyák, Renáta Minorics, István Zupkó

**Affiliations:** 1 Institute of Pharmacodynamics and Biopharmacy, University of Szeged, H-6720 Szeged, Hungary; 2 Department of Inorganic, Organic and Analytical Chemistry, University of Szeged, H-6720 Szeged, Hungary; 3 Laboratory of Functional Genomics, Biological Research Centre, H-6726 Szeged, Hungary; 4 Department of Medical Chemistry, University of Szeged, H-6720 Szeged, Hungary; 5 Institute of Physics, University of Pécs, H-7622 Pécs, Hungary; 6 Department of Theoretical Physics, University of Szeged, H-6720 Szeged, Hungary; 7 Interdisciplinary Centre of Natural Products, University of Szeged, H-6720 Szeged, Hungary

**Keywords:** 13α-estrone and cervical carcinoma, antiproliferative, anti-invasive, apoptosis, tubulin-microtubule system

## Abstract

Cervical carcinoma is one of the most frequent malignant gynecological cancers in women of reproductive age. Because of the poor tolerability of currently available chemotherapeutic agents, efforts have been focused on developing innovative molecules, including steroids, that exert antineoplastic effects with a better safety profile. In addition to their endocrine properties, certain estrogens exhibit additional biological activities, such as antiangiogenic and anticancer effects. Based on previous studies, the antineoplastic properties of 13α-estrone sulfamate derivatives (13AES1-3) were investigated, and the mechanism of action for the most promising compound 13AES3 was explored. Based on their effects on the viability of different human adherent gynecological cancer cells, the SiHa cervical cell line was used for mechanistic experiments. The most active analog 13AES3 was shown to exert considerable proapoptotic effects, as evidenced by a colorimetric caspase-3 assay and fluorescent double staining. It also elicited antimigratory and anti-invasive effects in a concentration-dependent manner, as evidenced by wound healing and Boyden chamber assays, respectively. Regarding their mechanism of action, 13AES derivatives were shown to inhibit tubulin polymerization, and computer simulations provided a possible explanation for the importance of the presence of the chlorophenyl ring on the estrane skeleton. 13AES3 is considered to be the first 13α-estrone derivative with a significant antineoplastic potency against SiHa cancer cells. Therefore, it might serve as a valuable lead molecule for the design of anticancer agents targeting cervical carcinomas.

## 1. Introduction

Cancer of the cervix uteri (cervical carcinoma) is the second most frequent malignant gynaecological cancers in women of reproductive age (following breast cancer), which seriously endangers women’s health and life worldwide [1]. Despite the successful implementation of HPV vaccination programs in developed countries, cervical carcinoma continues to be highly prevalent in Europe, with an estimated 58,373 cases and more than 20,000 deaths annually, thus it is still a significant public health issue [2].

The recognition rate of invasive cervical carcinomas has highly improved since the discovery of human papillomaviruses (HPV), identified as the principal etiological agent of these malignancies in 1983 when HPV16 was isolated from the DNA of a biopsy specimen taken from an invasive form of cervix cancer [3].

Following two decades of enormous efforts, the first safe and effective, quadrivalent prophylactic HPV vaccine was approved and introduced into clinical practice in 2006, which was effective against HPV6, -11, -16, and -18. The next product, a bivalent vaccine targeting HPV16 and -18 was approved a year later [4]. The latest product, a nonavalent vaccine, approved in 2015, is effective against nine types of HPV, including HPV6, -11, -16, -18, -31, -33, -45, -52, and -58 [5]. According to the American Joint Committee on Cancer (AJCC), approximately 13% of cervical cancer patients are diagnosed at advanced stages with an unfavorable prognosis. The median survival rate for metastatic cervical carcinoma patients is relatively low, estimated at 8–13 months. The five-year-survival rate of metastatic cervical carcinoma is 16.5%, compared to 91.5% for localized non-metastatic cancer of the cervix uteri [6]. 

Although many studies emphasize the implicating role of estrogens and their ERα receptors in various tumors, their association with cervical cancer is not well understood. Until now, the most apparent finding linking estrogens to cervical cancer comes from several recent studies on transgenic mice models, concluding that estrogens, along with their nuclear receptors, promote cervical cancer in the presence of HPV oncogenes [7,8,9]. HPV oncogenes (proteins E6 and E7) were found to come under the transcriptional control of the human keratin 14 promoter, which directs their expression to stratified squamous epithelium. As a result, it was concluded that HPV-transgenic mice could develop spontaneous tumors, primarily in the skin. However, when the mice were treated with 17β-estradiol, they began to develop cervical cancer, suggesting that cervical cancer is estrogen-dependent [10,11].

Since most chemotherapeutic agents have undesirable side effects and resistance may also limit therapeutic success, studies have focused on developing anticancer agents with better tolerability and safety profiles [12]. Using the steroid skeleton for designing new anticancer agents against cervical carcinomas is a promising approach, partly because of the advantages of this structural framework, as well as because these compounds tend to exhibit complex biological activities other than the classical hormonal effects [13,14]. Regarding the design of innovative anticancer molecules, an attractive feature of the steroid backbone is that even minor modifications of the steroid molecule may induce significant changes in the pharmacological profile [15]. 

**Figure 1:**
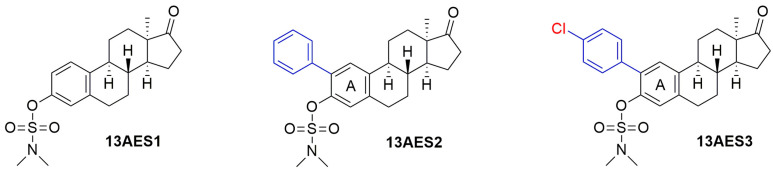
Structure of 3-*O*-sulfamoyl-13α-estrone derivatives (13AES1–3) possessing antiproliferative activities.

We have recently reported on the synthesis and pharmacological investigations of core-modified 13α- or D-secoestrone derivatives that lack hormonal activity [16,17,18,19,20,21,22,23,24]. The opening of ring D or the epimerization of C-13 yields estrone derivatives with low affinity for nuclear estrogen receptors [25,26]. Several core-modified compounds have been shown to display substantial antiproliferative activities against human cancer cell lines of gynecological origin [15]. In particular, we have demonstrated that the position, specific nature, size, and polarity of the new substituents introduced into the molecule considerably determine the anticancer properties of the new derivatives. The mechanism of antiproliferative action of some of our core-modified estrones relies on their direct effects on the tubule-microtubule system, which results in an increased rate of tubulin polymerization [16,18,22]. Based on literature data, establishing an *O*-sulfamate pharmacophore on the 3-OH function of the estrane core might lead to microtubule-disrupting properties, as well as proapoptotic and antiangiogenic actions. Certain estrone sulfamates possess anticancer activity on triple-negative breast cancer cells as well [27]. We have recently demonstrated that the introduction of a sulfamoyl moiety at the 3-OH or C-2–phenylation of 13α-estrone (13AES1–3, Figure 1) results in marked cell growth-inhibitory action against breast, ovarian and cervical cancer cell lines [28]. The 2-(4-chlorophenyl)-3-*O*-sulfamoyl derivative (13AES3) was found to be superior in terms of its antiproliferative activity against the HPV16-positive SiHa cell line. It should be emphasized that this HPV16-positive type is the most common form of invasive cervical cancer. The cell growth-inhibitory action of 13AES3, displayed at low micromolar concentrations, makes it a promising compound for the design of estrone-based, potent anticancer agents lacking estrogenic activity. Evaluating the mechanism of its antiproliferative action would be of great interest.

## 2. Results

### 2.1. Antiproliferative Properties of 13AES Compounds

The tested 13AES compounds exerted considerable antiproliferative activity on the cell line panel examined (including those for which our findings were published previously, and newly examined breast (T47-D) and cervical (C33A) cancer cell lines, see Table 1). In general, 13AES3 was found to be the most effective compound, and HPV-related cervical cancer cell lines (HeLa and SiHa) were found to be the most sensitive cancers with low micromolar IC_50_ values. Regarding tumor selectivity, all compounds exerted substantially less effect on NIH/3T3 fibroblasts, and the IC_50_ value of 13EAS3 was roughly one order of magnitude higher on these NIH/3T3 cells than on sensitive cervical cancer cells. Based on these findings, the SiHa cell line was chosen for additional cell-based investigations.

### 2.2. AES3 Induced Cell Cycle Disturbance

**Figure 2:**
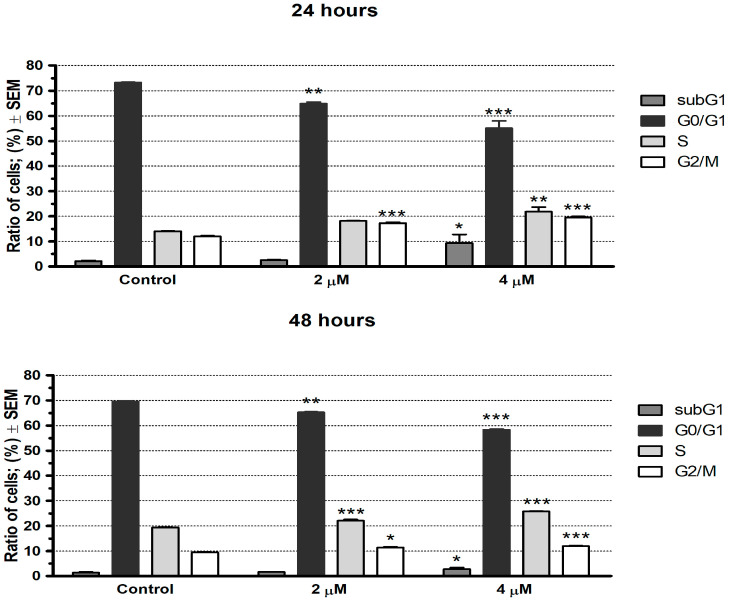
Cell cycle analysis by flow cytometry. 13AES3 induced cell cycle disturbances, characterized by an increased rate of the S and G2/M cell populations at the expense of a reduced rate of G0/G1 cells in SiHa cell lines. The upper and lower panels show the effects of 13AES3 on cell cycle phases at 24 and 48 h post-treatment, respectively. *, **, and *** indicate significance at *p* < 0.05, *p* < 0.01 and *p* < 0.001, respectively.

13AES3 was found to induce cell cycle disturbances at concentrations of 2 and 4 µM, characterized by a significant decrease in the proportion of cells in the G0/G1 phase and a noticeable accumulation of cells in the G2/M and S phases, in a concentration-dependent manner (Figure 2). Additionally, a pronounced increase in the hypodiploid subG1 cell population was noticed, particularly when treated with the higher concentration of 13AES3 (4 µM). The levels of these changes were comparable at 24 and 48 h of exposure to the test compound.

### 2.3. Morphological Alterations Induced by 13AES3 

**Figure 3:**
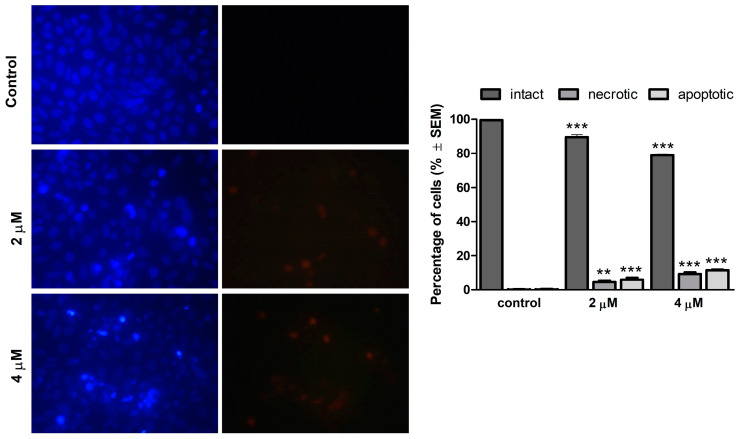
Morphological changes of SiHa cells after 24 h of exposure to 13AES3, visualized by HOPI double staining. A significant decrease in viable cells, as well as a remarkable increase in both necrotic and apoptotic cell populations, were induced by 13AES3. **Left panel**: representative fluorescent images (10× magnification); **right panel**: percentages of viable, necrotic, and apoptotic cell populations. ** and *** indicate significance at *p* < 0.01 and *p* < 0.001, respectively.

Based on the detectable elevation of the subG1 cell population in cell cycle analyses, the proapoptotic effect of 13AES3 could be proposed. Therefore, fluorescent double staining (HOPI) was performed to obtain further evidence for this property. Changes in cell morphology and membrane integrity were observed in SiHa cells at 24 h post-treatment. Fluorescent images of treated samples demonstrated a remarkable decrease in the viable, intact cell population, as well as a significant elevation in apoptotic cells emitting light blue fluorescence due to DNA condensation, and necrotic cells emitting red fluorescent due to damaged cell membrane (Figure 3). The increase in both apoptotic and necrotic cell populations exhibited concentration dependency.

### 2.4. Determination of Caspase-3 Activity

**Figure 4:**
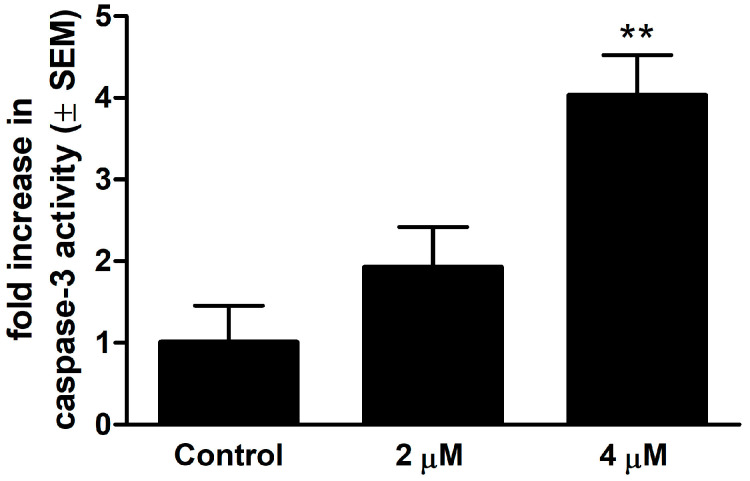
The proapoptotic property of the test compound was confirmed by assessing caspase-3 activity on SiHa cells at 24 h post-treatment. At a concentration of 4 μM, 13AES3 induced a 3-fold increase in caspase-3 enzyme activity compared to untreated control cells. ** indicates significance at *p* < 0.01.

Colorimetric measurement of caspase-3 enzyme activity showed a significant and concentration-dependent elevation after 24 h of exposure to 13AES3, indicating that the caspase cascade had been activated (Figure 4). While no significant effect was detected when the test compound was applied at lower concentration (2 µM), its solution of higher concentration (4 µM) induced a more than three-fold increase in caspase-3 enzyme activity compared to untreated controls.

### 2.5. Effects of 13AES1-3 Compounds on Tubulin Polymerization

**Figure 5:**
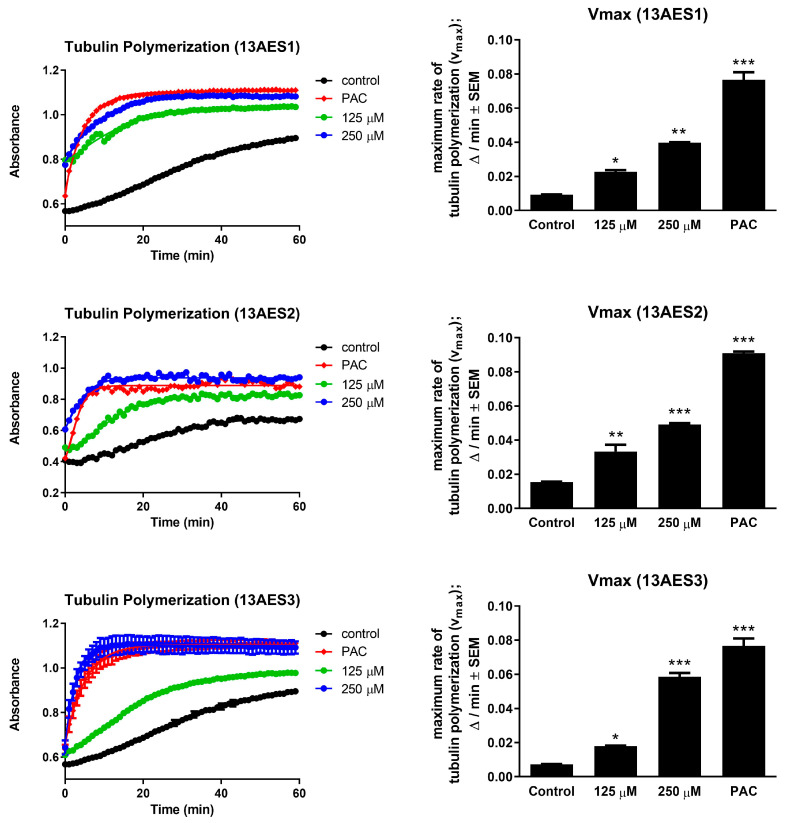
Tubulin polymerization assay. **Left panels**: trends of tubulin polymerization compared to the positive control (paclitaxel, PAC, 10 µM) and to vehicle. **Right panels**: calculated maximum rates of tubulin polymerization (Vmax). *, **, and *** indicate significance at *p* < 0.05, *p* < 0.01, and *p* < 0.001, respectively.

For a wider insight into the actions of 13AES1-3 compounds on tubulin polymerization, all were assayed using a cell-free system. Relatively high concentrations (125 and 250 µM) of the test compounds were applied, based on the manufacturer’s instructions and the IC_50_ values. Our results indicate that both higher and lower concentrations of all compounds showed a significant acceleration in tubulin polymerization compared to untreated control samples. The calculated maximum values of tubulin polymerization rate (Vmax) were statistically significantly increased by the test compounds at 250 µM concentration compared to untreated controls (Figure 5). The increase in polymerization rate was the highest with 13AES3. However, these changes were much lower than those detected with the reference agent paclitaxel. 

### 2.6. Molecular Modeling

Molecular modeling for all three compounds (13AES1-3) was carried out to simulate the theoretical binding preference order of the selected compounds as the primary outcome, which showed a good correlation with biological activity. Based on our findings using the umbrella sampling (US) method, ligand 13AES3 had the strongest interaction with the binding sites (−12.5 kcal/mol), whereas the other two compounds (13AES1 and 13AES2) were characterized by quite similar binding free energy values (−5.2 kcal/mol and −6.5 kcal/mol, respectively). Molecular mechanics/generalized Born surface area (MM/GBSA) calculations supported a very similar conclusion: 13AES3, 13AES2, and 13AES1 showed −69.38 ± 2.14 kcal/mol, −50.85 ± 2.85 kcal/mol, and −42.76 ± 2.35 kcal/mol ΔG values, respectively. These findings confirmed the US results and allowed further analysis of ligand–protein interaction as each MM/GBSA calculation was based on 500 ns long explicit water simulations. The outcomes of these analyses are presented in Figures S1–S3 in the Supporting Information. 

**Figure 6:**
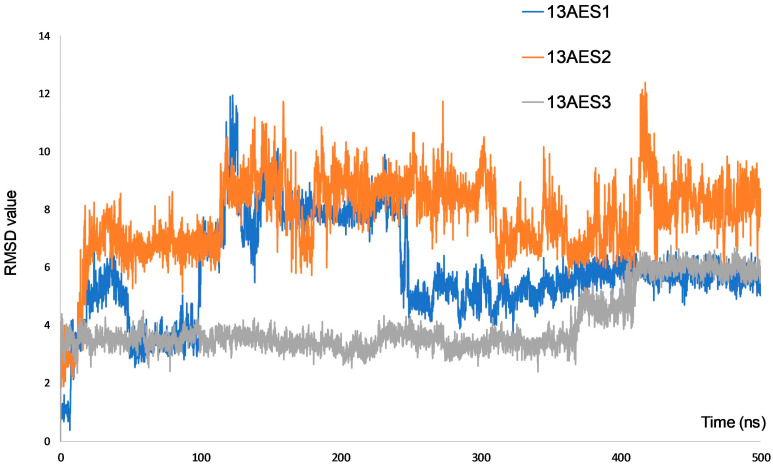
RMSD curves of the three 13AES ligands examined.

In the case of a ligand with strong binding ability, stability within the binding pocket is an important issue, which can be characterized by the RMSD curve. Large fluctuations in RMSD values indicate a less stable and, therefore, weaker binding. Figure 6 shows the RMSD curves for each ligand along the 500 ns trajectories. It is evident that ligands 13AES1 (blue line) and 13AES2 (orange line) show more extensive RMSD changes, whereas ligand 13AES3 (grey line) is characterized by more moderate RMSD fluctuations.

These findings align with our experimental results, as both 13AES1 and 13AES2 showed less pronounced biological activity than 13AES3 at a concentration of 30 μM. In the Supporting Information (Figures S1–S3) we provide a summary of the most essential ligand–protein interactions per residue along the 500 ns single-strand MD runnings. According to the analysis, there are two stable connections for ligand 13AES3 (T276, R369), which residues can have a crucial role in the higher ΔG value of this ligand. Interestingly, the number of interacting residues is higher for the ligands with weaker interactions (13AES1, 13AES2), but stability (quantified by the interactions fraction) was found to be the highest for ligand 13AES3.

### 2.7. 13AES3 Demonstrated a Remarkable Antimigratory Effect

**Figure 7:**
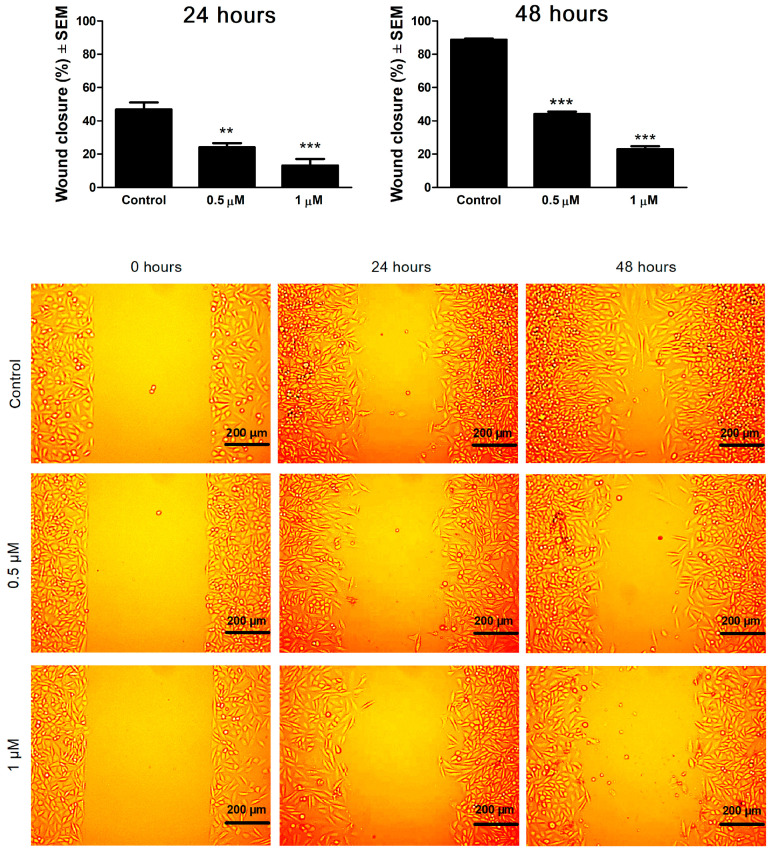
Cell migration (wound healing) assay. **Upper panels**: effects of 13AES3 on SiHa cell migration at 24 and 48 h post-treatment. **Lower panel**: representative images of the antimigratory effects of 13AES3 at 0, 24 and 48 h post-treatment. ** and *** indicate significance at *p* < 0.01 and *p* < 0.001, respectively.

To examine the antimigratory activity of 13AES3, a wound healing assay was conducted in serum-reduced medium (2% FBS) using special silicon inserts as an in vitro model of wound induction. Image-based data were analyzed to calculate the cell migration rate (percentage of wound closure) in both samples treated with the test compound and untreated controls. Based on the changes detected at 24 and 48 h post-treatment compared to untreated controls, 13AES3 was found to exert a concentration- and time-dependent antimigratory effect (Figure 7).

### 2.8. 13AES3 Demonstrated Substantial Anti-Invasive Activity

**Figure 8:**
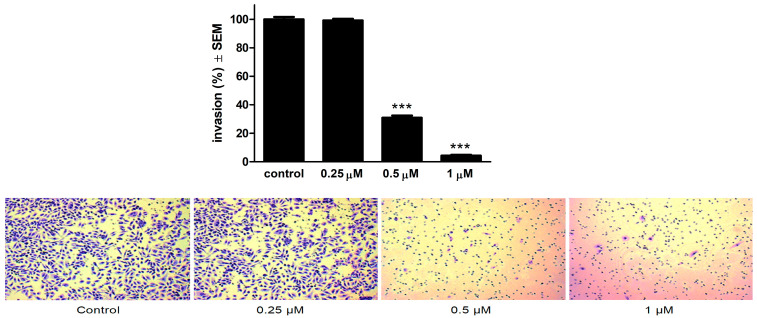
13AES3 elicited a significant anti-invasive effect on SiHa cells, at subantiproliferative concentrations. **Upper panel**: anti-invasive effect of 13AES3 at 24 h post-treatment. *** indicates significance at *p* < 0.001. **Lower panel**: microscopic images showing the density of invasive cancer cells after crystal violet staining (10× magnification).

A Boyden chamber assay was performed to determine the anti-invasive capacity of 13AES3. In addition, image-based data were obtained for each sample to determine the percentage of invading cancer cells by calculating the total number of invading cells in each well. Compared to untreated control samples, 13AES3 was found to exert a substantial and concentration-dependent anti-invasive effect at a concentration of 0.5 and 1 μM (Figure 8). In contrast, the lowest applied concentration (0.25 μM) did not induce any noticeable changes.

## 3. Discussion

It is widely accepted that estrogens and estrogen receptors play an essential role in the progression of numerous cancer types. Besides their essential role in the female reproductive system, estrogens exert important physiological effects in other body systems as well, including the brain, breast, bone, and the cardiovascular system via their receptors (ERα and ERβ) [29,30]. As part of the female reproductive tract, the uterine cervix seems to be highly sensitive to estrogens, particularly during the mid-follicular phase of the menstrual cycle when cervical epithelial cells begin to proliferate under estrogen influence, which may result in the development of hyperplastic epithelium. This process might be linked to the profound expression of ERα in the cervix, which is essential for the dynamic changes of the cervical epithelium [31,32]. Despite the controversial role of estrogens in cervical cancer progression, numerous steroid-based synthesized compounds lacking hormonal activity proved to be potent antiproliferative agents. Therefore, the present study aimed to investigate the in vitro anticancer effects of the newly synthesized 13AES3 and its mechanism of action.

Antiproliferative agents of steroidal origin are of great value owing to their substantial inhibitory effects against different cancer cell lines. Our previous studies focused on examining the in vitro antiproliferative activity of several sets of newly synthesized steroids developed with modifications on rings D and A. Some of these compounds exerted considerable antiproliferative effects against adherent gynecological cancer cell lines [16,18,33,34].

Regarding our current test substances, the chlorophenyl derivative (13AES3) displayed considerable growth inhibitory effect against cancer cell lines of gynecological origin. In addition, 13AES3 was found to be especially active against HPV-positive cervical cancer cells HeLa and SiHa with low micromolar IC_50_ values. HPV18-positive HeLa cells were slightly more sensitive than the SiHa cell line containing HPV16. However, the latter was reported to be more common in human cervical cancer samples and more frequently involved in the pathogenesis of other diseases, such as seborrheic keratosis or oropharyngeal and penile cancer [35,36]. Based on these considerations, the SiHa cell line was used for additional mechanistic studies. 

No modification at the C2 position was done in compound 13AES1, whereas the 2-phenyl functional group was substituted in both 13AES2 and 13AES3. The antiproliferative potential of C-3-O modified 13α-estrone derivatives greatly depends on the nature of the introduced moieties. Moreover, 4-chlorophenylation together with N,N-dimethyl pharmacophore substantially improved the antiproliferative potential against HPV16-positive cervical cells (SiHa). Therefore, this substitution pattern could improve the antiproliferative activity of 13AES3. In addition to the substantial inhibition of SiHa cells, investigating tumor selectivity patterns was the next necessary step. The non-cancerous fibroblast cells (NIH/3T3) were moderately inhibited by 13AES3 even at the higher concentration and were much lower than the reference agent cisplatin (Table 1). These data suggest that our test substance has tumor selectivity.

Cell cycle analysis was conducted to investigate the mechanism of action of 13AES3. Cell cycle disturbances induced by the test compound were detected, characterized by a decrease in the cell population in the G0/G1 phase and an increased proportion of cells in the G2/M and S phases. Moreover, an increase in the ratio of the hypodiploid cell population was detected, suggesting the ability of 13AES3 to induce apoptotic cell death. The proapoptotic property of our test substance was further confirmed by colorimetric determination of caspase-3 enzyme activity and fluorescence microscopy.

Microtubules are dynamic filamentous cytoskeletal proteins, considered as important therapeutic targets in cancer patients [37]. Agents targeting tubulin monomers are classified into microtubule-stabilizing and destabilizing drugs. The first group, including for example, paclitaxel, enhances tubulin polymerization, whereas microtubule destabilizing agents, such as *Vinca* alkaloids, inhibit or reduce tubulin polymerization [38]. In the field of antineoplastic chemotherapy, tubulin binding agents represent a class of anticancer drugs with a broad spectrum of biological activities. Presumably, these agents, also known as antimitotic drugs, interfere with cell division, specifically affecting the mitotic spindle of cancer cells [39]. Therefore, a cell-free tubulin polymerization assay was conducted to evaluate the effects of our test substance on the microtubular system. All three 13AES agents induced a significant acceleration of tubulin polymerization, which agrees with the disturbed cell cycle and the marked increase in the proportion of cells at the G2/M detected in the cell cycle analysis. 

Computational simulation results suggest that the chlorophenyl ring of 13AES3 at position 2 helps to stabilize the binding pose of the sterane skeleton, explaining the stronger interaction with β-tubulin. Namely, increased hydrophobic interaction between the chlorophenyl ring of ligand 13AES3 and the phenyl ring of F272 residue was revealed (see Figure S3). It is important to note that a hydrophobic interaction between ligand 13AES2 and the phenyl ring of F272 was also established. However, this hydrophobic interaction was less significant and was not exclusively formed between the phenyl rings. The phenyl ring of F272 partially interacted with the aromatic ring A of the estrane skeleton.

**Figure 9:**
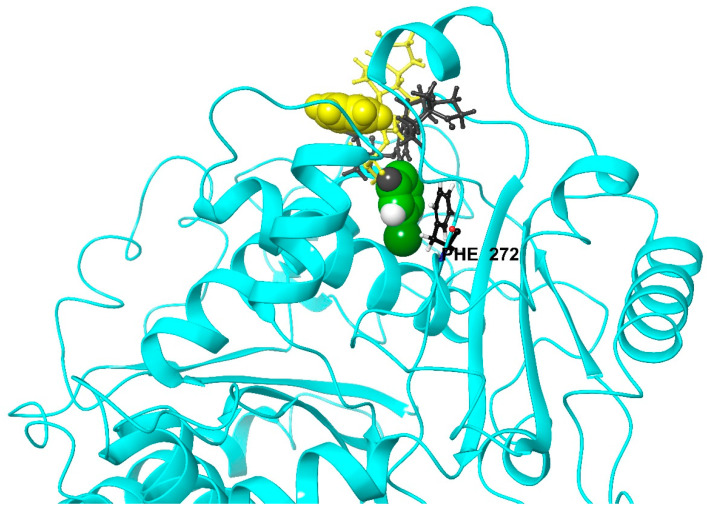
Representative binding positions of ligands 13AES2 (yellow) and 13AES3 (green). The blue ribbon represents the protein backbone of β-tubulin, and the atoms of the two phenyl rings at position 2 are represented by Van der Waals balls. The Phe272 residue of special interest is also shown in a stacking geometry for ligand 13AES3.

Consequently, the sterane scaffold was less oriented for 13AES2, and thus it could not establish a stable link to the protein through the 17-keto and 3-*O*-sulfamoyl groups. Furthermore, visual inspection of the trajectories suggested that in the presence of a halogen atom, the chlorophenyl ring could penetrate deeper into the binding pocket, which considerably restricted the ligand’s mobility. This restriction is demonstrated in Figure 9, where two representative geometries of ligands 13AES2 and 13AES3 are presented. Figure 9 also illustrates the relative position of the F272 residue, which is located deeper in the binding pocket, so an increased interaction with this residue suggests a deeper (therefore a stronger) binding pose. Thus, it can be concluded that the presence of an unsubstituted phenyl ring (13AES2) promotes the hydrophobic connection, but it is insufficient; increased hydrophobicity must be achieved, which could be realized by the introduction of a halogen atom.

Based on epidemiological data, the invasive form of cervical cancer is highly prevalent, and ranks the fourth most common type of cancer in women, following breast, colon, and lung cancers, with 528,000 new cases per year worldwide. Metastases of cervical carcinomas develop either by the hematogenous or the lymphatic route. Patients with hematogenous metastases tend to have a lower survival rate than those with lymphatic metastases [40,41,42,43]. As proof of the antimetastatic property of our test compound, 13AES3 inhibited the migration and invasion of SiHa cells significantly and in a concentration-dependent manner. Both wound closure, an indicator of migration, and invasion were substantially inhibited by a submicromolar concentration of 13AES3. Moreover, it was demonstrated to enhance tubulin polymerization, manifested in cell cycle disturbances. Since tubulin dynamics have a crucial role in the mobility of cancer cells, a tubulin-disrupting agent may exert antimetastatic effects besides direct cytotoxicity [44]. Based on these findings, an outstanding antimetastatic potential of 13AES3 may be proposed.

## 4. Materials and Methods

### 4.1. Chemicals and Cell Culture

The tested steroidal sulfamates (13AES1-3, Figure 1) were synthesized as described previously in detail [28]. For all in vitro experiments, 10 µM stock solutions of the test compounds were prepared with dimethyl sulfoxide (DMSO). Human cervical cell lines SiHa (HPV16-positive squamous carcinoma) and C33A (HPV-negative carcinoma) were purchased from the American Tissue Culture Collection (ATCC, Manassas, VA, USA), while all other cell lines were obtained from the European Collection of Cell Cultures (Salisbury, UK). Cells were cultured in a minimal essential medium supplemented with 10% fetal bovine serum, 1% non-essential amino acids, and 1% of antibiotic-antimycotic mixture in humidified atmospheric air containing 5% CO_2_ at 37 °C. All cell culture chemicals were obtained from Lonza Group Ltd. (Basel, Switzerland). 

### 4.2. Antiproliferative Activity

The antiproliferative properties of the test compounds were determined on a panel of human gynecological cancer cell lines and were reported previously [28]. These previous results were now completed with assays on cells from breast (T47-D) and cervical (C33A) cancer using the same protocol. T47-D and C33A cells were seeded onto 96-well microplates at a density of 5000 and 10,000 cells/well, respectively, and were incubated overnight under standard cell culture conditions. Two concentrations (10 and 30 μM) of the test compounds were used as the first step of the investigation. In case of substantial activity (i.e., growth inhibition >50%), the assays were performed with a set of dilutions, and IC_50_ values were calculated by the GraphPad Prism 5 software (GraphPad, San Diego, CA, USA). All in vitro antiproliferative experiments were repeated twice with at least five parallel wells. 

### 4.3. Cell Cycle Analysis

Cell cycle analysis was conducted to measure the cellular DNA contents of cells using flow cytometry. First, SiHa cells were seeded onto 24-well plates at a density of 80,000 cells/well and were incubated overnight. The cells in each well were then treated with 50 µL of fresh medium containing the desired concentrations of 13AES3 (2 and 4 µM) and were incubated for 24 or 48 h. Next, the cells were washed with PBS and were harvested by trypsin, the cell suspensions were centrifuged (1400 rpm, 5 min, room temperature), and the supernatants were carefully removed. Finally, the DNA content of the cells was stained by adding 300 µL of PI solution (10 µg/mL propidium iodide, 0.1% Triton-X, 0.1% sodium citrate, and 10 µg/mL RNase-A dissolved in PBS), and the samples were stored in the dark at room temperature for 30 min. Eventually, the cells were analyzed by a FACSCalibur flow cytometer, where at least 20,000 events per sample were evaluated for each analysis. Data were analyzed by the ModFit LT 3.3.11 software (Verity Software House, Topsham, ME, USA). Untreated cells were used as a control. The hypodiploid (subG1) cells were regarded as apoptotic cells. Cell cycle analysis experiments were repeated three times with two parallel samples of each condition.

### 4.4. Hoechst 33258/Propidium Iodide Fluorescent (HOPI) Staining

Fluorescent staining was performed to detect the necrotic and apoptotic morphological changes induced by 13AES3 in treated cells. SiHa cells were initially seeded onto a 24-well plate at a density of 50,000 cells/well and were incubated overnight. The cells were then treated with the desired concentrations of the test compound and were incubated for 24 h under the cell culturing conditions described above. Following treatment, the cells were stained with a medium containing lipophilic Hoechst 330258 (HO, 5 µg/mL) and hydrophilic propidium iodide (PI, 1 µg/mL) and were incubated in dark for 60 min. Then, the medium was refreshed, and images (five per each condition) were taken using a Nikon Eclipse TS100 fluorescent microscope (Nikon Instruments Europe, Amstelveen, The Netherlands) equipped with appropriate filters (HO: excitation: 360/40 nm bandpass filter, emission: 460/50 nm bandpass filter and 400 nm dichromatic mirror; PI: excitation: 500/20 nm bandpass filter, emission: 520 nm longpass filter, and 515 nm dichromatic mirror). Cell nuclei emitting fluorescence were counted in each sample (100–300 cells/image depending on the concentration of the test substance), and the proportions of intact, necrotic, and apoptotic cells were expressed as percentages of total cell count.

### 4.5. Caspase-3 Activity Measurement

The proapoptotic activity of 13AES3 was assayed by a commercially available colorimetric caspase-3 kit (Abnova, Taipei, Taiwan). First, SiHa cells were seeded onto the culture flasks (1 × 10^7^ and 1.5 × 10^7^ control and treated cells, respectively) and were incubated overnight. Next, the cells were treated with the desired concentration of the test compound (2 or 4 µM) and were incubated for 24 h under the cell culture conditions described above. The cells were scraped by a cell scrubber, counted, washed with PBS, and centrifuged at 3000 rpm, 4 °C, for 15 min. The pellets were then re-suspended in an appropriate lysis buffer (100 μL/107 cells) and were incubated on ice for 20 min. Then, the lysates were centrifuged at 600× *g*, 4 °C, for 15 min. Finally, cell lysates of each sample with substrate and assay buffer were pipetted into a 96-well plate and were incubated overnight according to the manufacturer’s instructions. Absorbance of the cleaved substrate is directly proportional to the amount of active caspase-3, and it was measured by a microplate reader at 405 nm (BMG Labtech, Ortenberg, Germany). In addition, changes in caspase-3 activity were detected by comparing the absorbance of treated samples to untreated controls. The experiment was performed with five parallel samples.

### 4.6. Tubulin Polymerization Assay

The effects of the test compounds on tubulin polymerization were assayed using a commercially available kit (Cytoskeleton, Denver, CO, USA) on a pre-warmed 96-well microplate. According to the manufacturer’s instructions, concentrations of the tested agents should be approximately 100-fold higher than their IC_50_ values; therefore, 13AES compounds were used in concentrations of 125 and 250 µM. The polymerization reaction was initiated by adding 100 µL of tubulin (3 mg/mL in 80 µM PIPES, pH 6.9) to each well. Measurement of absorbance of the samples was started immediately and was repeated at each minute, performed at 340 nm using a 60 min kinetic measurement protocol. Paclitaxel (10 µM) was included as a reference agent. Maximum reaction rate (Vmax) was regarded as the highest difference between two consecutive moving averages of three points. Tubulin polymerization experiments were performed twice with two parallel samples. 

### 4.7. Computational Simulations

Molecular dynamics (MD) simulations were performed with the Desmond module of the Schrodinger suite [45] based on the crystal structure of taxol-stabilized microtubule protein complex (PDB entry: 5SYF) [46]. The Protein Preparation Wizard tool from the Schrodinger’s Maestro GUI was applied to generate a proper starting geometry and pH state for the initial complex [47]. Next, a short (5-ns-long) relaxational MD running was performed, and the last frame of the MD trajectory was applied to provide the target geometry for the docking simulations. Docking calculations were accomplished with the Glide program from the Schrodinger suite using the extra precision (XP) protocol [48]. The docking grid was generated in the 10 Å environment of the taxol binding site, which area was selected as the target region in our previous studies [18,22]. In those papers, it was pointed out that this area could serve as a binding pocket for ligands with a sterane skeleton. Following the docking calculations, the binding pose with the best Glide-score was considered as the starting structure of each ligand, and 500-ns-long single-strand MD simulations were performed with the Desmond module of the Schrodinger suit. The binding free energy (ΔG) of protein–ligand complexes was calculated according to the Molecular Mechanical (MM) level/generalized Born surface area (MM/GBSA) method using 5000 dynamic trajectory points [49] in each case. OPLS4 force field and simple point charge (SPC) water model were applied, and the MM/GBSA calculations were determined by the “thermal_mmgbsa” python script from the Schrodinger suite. Mean ΔG values of a complex (and standard error of the means, available in the Supporting Information) were determined by bootstrap calculations using an in-house script, where 5000 bootstrap iterations were performed with eight randomly selected data points from the calculated ΔG values. All figures were prepared with the Maestro program, the GUI part of the Schrodinger program package.

Another widely accepted ΔG calculating technique is the so-called Umbrella Sampling (US) method. A typical US computation starts with a pulling phase, where the ligand is forced to leave the binding pocket, followed by several short MD simulations (umbrella windows) along the pulling trajectory with constrained ligand–protein distance. Our US pulling calculations were always started from the same docking complexes as the MM/GBSA computations. The ff14SB and GAFF2 force field were applied for the protein and the ligands, respectively, in combination with the TIP3P explicit water model [50,51,52]. The pulling time was 1 ns long, and 200 ps equilibration was performed in each umbrella window. These umbrella windows were applied when the weighted histogram analysis method (WHAM) [53] provided the final potential of the mean force (PMF) curve. In our complexes, the longest protein–ligand constrain distance was around 35–40 Å, and the restraint potential was set to 0.75 kcal/mol. All simulations were performed at 310K and 0.15M physiological salt concentration. All the US calculations were performed with the Amber20 program package [54].

It is important to note that both ΔG calculation methods are usually applied to determine relative ΔG values, therefore, the absolute values have less meaning, and it is better to focus on the binding preference order of the ligands. Finally, representative structures were generated by clusterization along the 500 ns trajectory, where the protein backbone (without the keto oxygen) was fitted and clustered into five clusters, from which the representative structure of the largest cluster was selected as the representative complex geometry.

### 4.8. Migration Assay

The effect of 13AES3 on cancer cell migration was determined by a wound healing assay using specific wound assay chambers (ibidi GmbH, Martinsried, Germany). First, SiHa cells were seeded onto each chamber of the inserts at a density of 50,000 cells/well and were incubated overnight at 37 °C with 5% CO_2_ to ensure proper cell attachment. The inserts were then gently removed, and the wells were washed twice with 5 mL of phosphate-buffered saline (PBS) to remove non-adherent cells and debris. Then, the cells were treated with fresh medium containing 2% fetal bovine serum (FBS) and the desired concentrations of 13AES3 (0.5 or 1 µM) and were incubated again for 24 or 48 h. The migration of cells toward the wound closure site was visualized using a phase-contrast inverted microscope (Nikon Eclipse TS100 microscope, Nikon Instruments Europe, Amstelveen, The Netherlands). Images were taken for each sample using a CCD camera (QImaging MicroPublisher Color RTV5.0, Teledyne Photometrics, Tucson, Arizona, USA) to assess the extent of wound closure. The percentage of cell migration was calculated in treated samples at different intervals (0, 24, and 48 h) by determining cell-free area, and these values were compared with untreated control samples assessed at the same intervals using the ImageJ software (National Institutes of Health, Bethesda, MD, USA). Migration assay experiments were repeated twice with at least four parallel samples.

### 4.9. Invasion Assay

The anti-invasive capacity of the test compound 13AES3 was assessed by a Boyden chamber assay using the BD BioCoat Matrigel Invasion Chamber with an 8 micrometer pore-size PET membrane containing a thin layer of Matrigel Basement Matrix (BD Biosciences, Bedford, MA, USA). First, SiHa cell suspensions prepared in serum-free medium containing the desired concentrations of 13AES3 (0.5 or 1 µM) were pipetted onto the membrane of the upper chamber. Medium with 10% FBS was used as a chemoattractant in the lower compartment. After 24 h of incubation, the supernatants were removed carefully, and the upper side of the membrane was cleaned gently from non-invading cells with a cotton swab soaked in PBS. Next, the membrane was washed twice with PBS and fixed with ice-cold 96% ethanol. Later, the invading cells were stained by crystal violet dye (1%) and were kept in dark for 30 min at room temperature. Lastly, images were taken (5 per insert) using a Nikon Eclipse TS100 microscope. Then invading SiHa cells were counted, and their number was compared to the untreated control. Invasion assay experiments were repeated three times with two parallel samples.

### 4.10. Statistical Analysis

Statistical evaluation of the results was performed by one-way analysis of variance followed by the Dunnett posttest, using the GraphPad Prism 5 software (GraphPad, San Diego, CA, USA). Data were expressed as mean values ± standard error of the mean (SEM).

## 5. Conclusions

Among the investigated 13AES analogs, the 2-(4-chlorophenyl)-13α-estrone sulfamate derivative 13AES3 exerted substantial antiproliferative and antimetastatic effects, and considerable proapoptotic properties were demonstrated against the HPV16-positive human cervical carcinoma cell line (SiHa). In addition to inducing cell cycle disturbances, it also induced alterations in cell morphology and membrane integrity. Concerning its antimitotic potentials, 13AES3 stabilized tubulin via increasing the rate of tubulin polymerization and possessing a strong protein–ligand interaction. To the best of our knowledge, 13AES3 is regarded as the first 13α-estrone derivative with such a high potency against the invasive cervical cancer cell line SiHa. Therefore, it might serve as a valuable lead molecule for the design of anticancer agents targeting cervical carcinomas.

## Figures and Tables

**Table 1:** Antiproliferative properties of 13AES1–3.

		Conc. (µM)	13AES1	13AES2	13AES3	Cisplatin
MCF-7 *	Inhibition (%)	10 μM	60.47 ± 2.62	61.11 ± 2.36	57.84 ± 1.56	53.03 ± 2.29
		30 μM	82.67 ± 1.15	75.83 ± 2.58	81.64 ± 2.61	86.90 ± 1.22
	Calculated IC_50_ (μM)		5.28	6.72	6.36	5.78
MDA-MB-231 *	Inhibition (%)	10 μM	15.48 ± 2.29	29.40 ± 0.71	35.66 ± 0.64	20.75 ± 0.81
		30 μM	42.14 ± 1.23	37.59 ± 0.70	65.31 ± 1.94	71.47 ± 1.20
	Calculated IC_50_ (μM)		n.d. **	n.d.	16.34	19.13
T47D (breast)	Inhibition (%)	10 μM	58.41 ± 3.31	39.46 ± 1.55	61.29 ± 1.16	40.76 ± 1.81
		30 μM	71.21 ± 2.19	71.31 ± 2.61	73.00 ± 0.96	59.96 ± 0.66
	Calculated IC_50_ (μM)		8.28	9.66	9.11	17.48
HeLa *	Inhibition (%)	10 μM	67.84 ± 0.86	62.78 ± 0.47	81.11 ± 0.67	42.61 ± 2.33
		30 μM	69.78 ± 1.08	69.39 ± 0.80	95.45 ± 0.80	99.93 ± 0.26
	Calculated IC_50_ (μM)		6.67	7.53	2.28	12.43
SiHa *	Inhibition (%)	10 μM	58.35 ± 0.66	48.91 ± 1.60	78.53 ± 2.53	88.64 ± 0.50
		30 μM	60.32 ± 1.09	55.37 ± 0.77	91.44 ± 0.94	90.18 ± 1.78
	Calculated IC_50_ (μM)		13.21	15.95	2.71	7.84
C33A	Inhibition (%)	10 μM	48.20 ± 2.84	22.25 ± 1.77	44.96 ± 0.44	67.41 ± 4.56
		30 μM	70.98 ± 1.65	61.64 ± 0.99	58.50 ± 1.43	92.21 ± 0.41
	Calculated IC_50_ (μM)		11.15	24.74	17.40	3.54
A2780 *	Inhibition (%)	10 μM	23.38 ± 1.50	31.83 ± 1.45	50.01 ± 1.04	83.57 ± 1.21
		30 μM	40.16 ± 2.09	41.57 ± 2.10	76.55 ± 1.01	95.02 ± 0.28
	Calculated IC_50_ (μM)		n.d.	n.d.	10.60	1.30
NIH/3T3 * (fibroblast)	Inhibition (%)	10 μM	23.98 ± 2.16	21.04 ± 1.09	34.27 ± 1.93	91.80 ± 0.39
		30 μM	44.12 ± 2.35	25.72 ± 2.72	50.57 ± 1.14	93.68 ± 0.20
	Calculated IC_50_ (μM)		n.d.	n.d.	26.56	2.70

* Previously published data [28]; ** n.d.: not determined.

## Data Availability

Data are available upon request.

## Supplementary Materials

The following supporting information can be downloaded at: https://www.mdpi.com/article/10.3390/ijms24076625/s1.
