# Supplementary material for: Investigation of the Antineoplastic Effects of 2-(4-Chlorophenyl)-13α-Estrone Sulfamate against the HPV16-Positive Human Invasive Cervical Carcinoma Cell Line SiHa
Source: Int J Mol Sci. 2023 Apr 1;24(7):6625. doi: 10.3390/ijms24076625 (PMC10095317; doi:10.3390/ijms24076625)
Supplement: Supplementary file 1 [file ijms-24-06625-s001.zip › ijms-2274353-supplementary.pdf]

Supporting Information

Investigation of the Antineoplastic Effects of 2-(4-Chlorophenyl)-13α-Estrone Sulfamate against the HPV16-Positive Human Invasive Cervical Carcinoma Cell Line SiHa

Hazhmat Ali, Péter Traj, Gábor J. Szebeni, Nikolett Gémes, Vivien Resch, Gábor Paragi, Erzsébet Mernyák, Renáta Minorics, István Zupkó

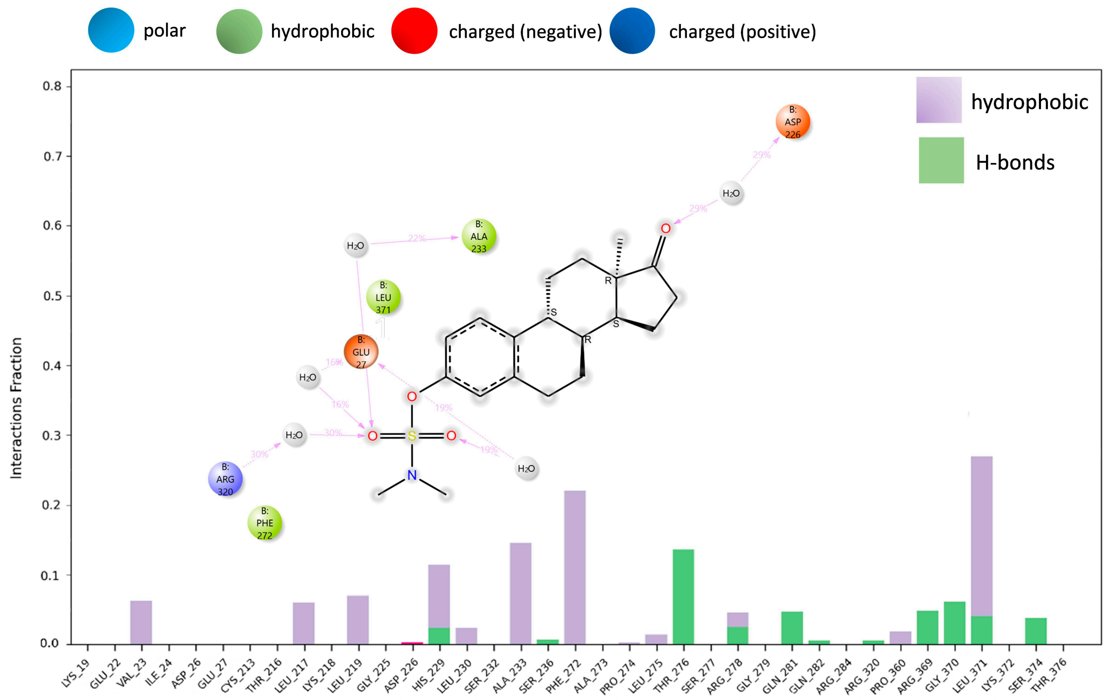

Figure S1. Ligand-Protein (LP) interactions along the 500 ns long trajectories of 13AES1.

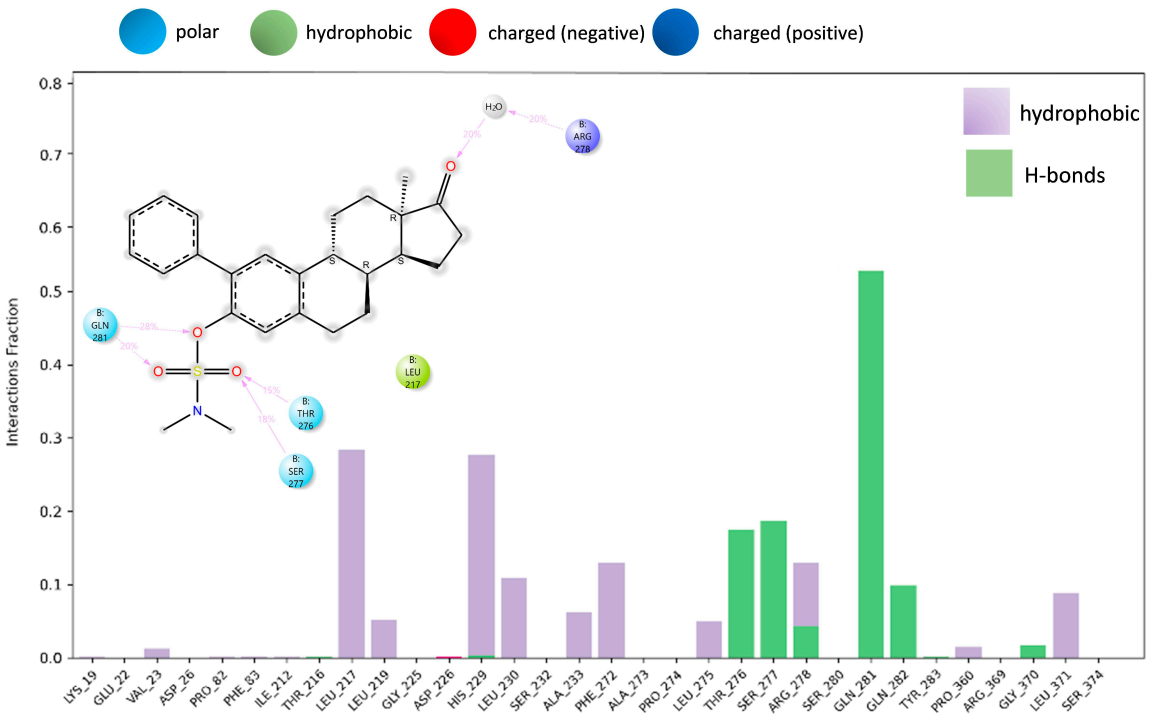

Figure S2. Ligand-Protein (LP) interactions along the 500 ns long trajectories of 13AES2.

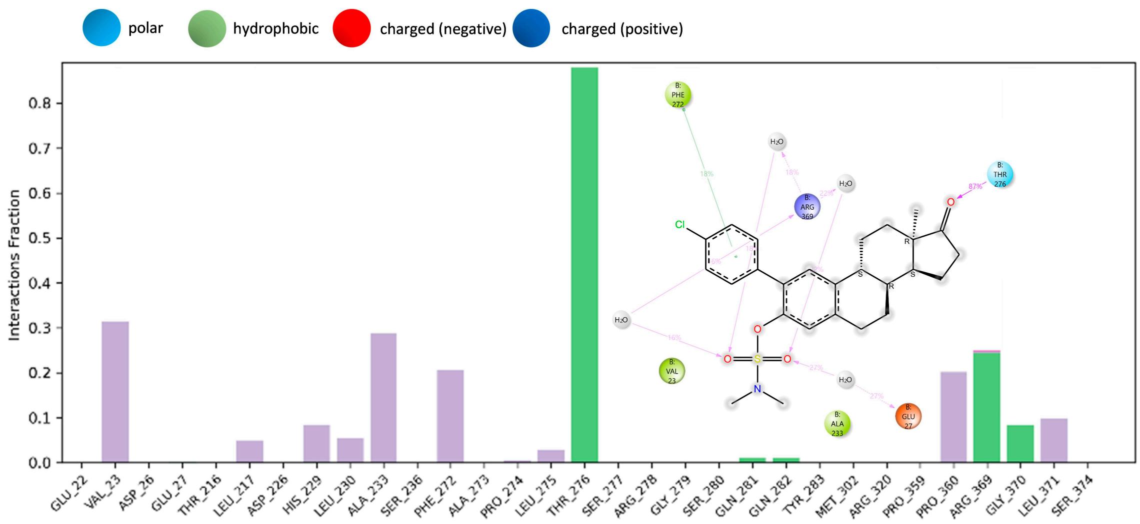

Figure S3. Ligand-Protein (LP) interactions along the 500 ns long trajectories of 13AES3.
